# Exploring associations between sleep duration and performance as well as heart rate variability in elite esports athletes

**DOI:** 10.1038/s41598-025-18228-y

**Published:** 2025-09-24

**Authors:** Peter Hoang, Mark Deppe, Brenden Alvarez, Alaina A. Klaes, John Billimek, Brian Y. Kim

**Affiliations:** 1 Department of Family Medicine, University of California, Irvine School of Medicine, Irvine, CA USA; 2 University of California, Irvine, Irvine, CA USA

**Keywords:** Sleep extension, Esports, Gaming performance, Heart rate variability, Oura ring, Health occupations, Medical research

## Abstract

Sleep is essential for overall well-being, influencing physical, psychological, and mental health. While research shows that increasing sleep enhances athletic performance, data on the relationship between performance, sleep, and stress response in esports athletes remains limited. Our study aims to explore sleep counseling and its associations on neurocognitive function, gaming performance, and heart rate variability in esports. We enrolled competitive Valorant esports athletes. Participants maintained their regular sleep schedule for four to six weeks, followed by an attempted four-week sleep extension phase. Health data were collected via a wearable device. We evaluated individual performance through neurocognitive tests and by analyzing participants’ weekly match statistics. The study revealed that participants did not significantly increase sleep duration (*p* = 0.265). We observed improvements in neurocognitive test reaction times (*p* < 0.01) but not gaming performance. Sleep counseling was also associated with improved heart rate variability (*p* < 0.05), though the effect size was small. Overall, this study highlights the difficulty of implementing behavioral sleep interventions in elite esports athletes and the need for alternative or more targeted approaches. While the intervention did not achieve significant sleep extension and therefore constrained causal inference, the study demonstrates the feasibility of a rigorous methodological framework for investigating sleep and performance in esports athletes.

**Supplementary Information:**

The online version contains supplementary material available at 10.1038/s41598-025-18228-y.

## Background

The increasingly popular field of electronic sports (esports) refers to organized video game competitions. Over the past decade, esports have evolved from small communities into a global phenomenon, attracting millions of players and spectators worldwide^1–4^. The global esports audience has experienced significant growth, with estimates indicating that the number of esports enthusiasts will range from 474 million in 2021 to 640 million in 2025^5^. Esports appeal to a wide demographic across different genders and age groups, although it typically engages younger audiences^6,7^. The financial growth of esports mirrors its rising popularity, with the global market projected to reach $1.8 billion in 2025, driven by increasing interest and financial incentives. Over the past few years, even colleges and universities have begun to invest in esports, as seen through the growing number of esports club teams and scholarships^6^.

As the esports industry grows, research on optimizing performance and understanding the lifestyle demands of professional players has been developing. One emerging area of interest is the impact of sleep quantity on overall performance. Sleep plays a vital role in physical, psychological, and mental health and offers numerous benefits, such as improving cardiac health, reducing stress levels, and improving memory consolidation and learning^8–11^. Sleep deprivation is common in many other sports, leading to decreased cognitive abilities and productivity^12,13^. Athletes are particularly prone to sleep issues due to intense training, travel, and competition demands^14,15^. While elite athletes report needing about eight hours of sleep, most fall short of this threshold^16^. Studies show that adequate sleep improves athletic performance, while insufficient sleep impairs recovery and increases injury risk in both adults and children^17–19^. Sleep interventions, such as sleep extension and naps, have been found to enhance performance more effectively than sleep hygiene education alone^16^. Similarly, professional esports athletes undergo rigorous and lengthy training schedules, which can often adversely affect physical and mental health^4,20^. Prior studies indicate that sleep deprivation can impair gaming performance by slowing reaction times and increasing the likelihood of lapses (i.e., failure to respond or reaction time exceeding 500 milliseconds)^21^. Another study on 27 esports athletes showed that gaming sessions elicit significant physiologic changes such as reduced heart rate variability (HRV) and increased blood pressure^22^. These changes are typically seen with an impaired physiologic stress response, suggesting esports athletes are at risk of chronic stress outcomes that can negatively impact their performance. As mentioned earlier, sleep extension has demonstrated improved physical and mental ability on subsequent days and may provide a solution to correct the suboptimal sleep habits in the esports population.

Studies on the relationship between sleep, stress adaptation, and esports performance are lacking compared to those conducted for many other sports. To the best of our knowledge, there are only two published reports that have specifically explored the effect of sleep extension or deprivation in esports athletes^23,24^. Gaining a better understanding of sleep and stress and their influence on esports performance will allow teams to tailor training schedules that avoid accumulating fatigue and stress while offering a competitive advantage. Our investigation focuses on elite esports athletes competing in Valorant, a fast-paced, strategy-intensive game requiring high cognitive decision-making, concentration, and reaction time^25–27^. As a result, they represent a population that is susceptible to sleep-related impairments. The primary objective of our study was to assess the impact of sleep education and efforts to extend sleep on gaming and neurocognitive performance. Our secondary measures of interest were assessing HRV as well as wearable device satisfaction and compliance throughout the study.

## Methods

### Study recruitment and participants

Participants were recruited using online platforms through social media (Facebook, Instagram, Twitter), Discord server, and email. Inclusion criteria included participants above the age of 18 and minimum Valorant rank level of Diamond (roughly the top 10% of users worldwide). We excluded those who could not periodically present in person to the on-campus esports facility and those who were already using the Oura Ring or similar ring-based wearable health devices. Participants received a $200 gift card after completion of the study. Informed consent was obtained from participants before initiating the study. All aspects of the protocol were conducted according to the ethical guidelines and standards established by the University of California, Irvine Institutional Review Board and the Declaration of Helsinki.

## Measuring health data

Our study used the Oura Ring Gen3 (Oulu, Finland), a consumer wearable device that passively captures biometric data. We selected this device based on its battery life, ease of use, ability to integrate with multiple mobile operating systems, and data exportation capabilities. The device is worn on the finger and contains infrared photoplethysmography (PPG), negative temperature coefficient sensors, and a 3-dimensional accelerometer to help collect data. The Oura Ring measures sleep duration using movement patterns from an accelerometer and skin temperature. Additionally, it captures nighttime HRV using the root mean square of the successive differences (rMSSD), which calculates the differences between consecutive heartbeats, squaring the differences, averaging the values, then taking the square root of the averaged squared differences. Several studies have validated this device against polysomnography and electrocardiography, both considered gold-standard diagnostic tools for assessing sleep and cardiac electrical activity, respectively^28–31^.

All subjects were provided with a wearable health device, instructed to download the associated mobile application, and guided on how to link the device to their mobile phones. Participants were advised to limit their time on the mobile application except to check device battery life and troubleshoot issues. Participants also consented to join the Oura Teams service, a secure platform which allows individuals to share their wearable data with researchers. Health data monitoring, data collection, and data storage were compliant with institutional security and privacy regulations.

## Online surveys

At the first data collection point (T_0_), participants completed a baseline demographic questionnaire, followed by a four-question survey at the study’s midpoint (T_1_) and conclusion (T_2_) assessing their satisfaction with the wearable device. Responses were graded on a 5-point Likert scale. Surveys were designed in English using an online cloud survey software, Qualtrics version 02.2024 (Provo, Utah, United States; https://www.qualtrics.com), and distributed anonymously.

## Study design

In this study, participants served as their own controls, with baseline data collected during an initial washout period while maintaining regular sleep patterns for four to six weeks. Neurocognitive testing was conducted with several online instruments (described below). Our wearable health device requires a minimum calibration period of two weeks. Notifications for the mobile application were turned off to prevent bias during this data collection period. Following the baseline period, participants returned for repeat in-person neurocognitive testing. At this time, T_1_, participants were counseled on sleep hygiene using a handout we developed and instructed to increase their sleep duration by at least one hour each night for four weeks. They were encouraged to use the mobile application associated with the device, which provides many metrics on sleep, identifies areas of improvement, and contains various tools and tutorials on sleep hygiene, relaxation, and breathing exercises. For detailed information regarding our handout, see the supplementary data. At the last data collection timepoint, T_2_, participants completed the final session of neurocognitive testing.

## Neurocognitive testing

Attention, reaction time, visuospatial processing (e.g., quickly interpreting complex 2D/3D game environments), and multitasking (e.g., monitoring teammates’ status, game alerts) are important domains in esports. The Psychomotor Vigilance Test (PVT), Simple Reaction Time Test (SRT), and Choice Reaction Time Test (CRT), all previously validated in the literature, were used as proxies to assess neurocognitive performance^32,33^. Collectively, they are reliable indicators of readiness and performance under varying sleep conditions and easy to administer. The PVT is considered a gold standard measure of fatigue-related deficits and objectively measures alertness and attention span^34–37^. Participants are tasked with rapidly responding to visual cues randomly shown at specific intervals and without incorrectly responding when no visual cue is present. The primary outcome of interest was mean reaction time. The SRT and CRT are often used to measure the speed of decision-making. It involves two components: (1) a simple reaction time task where a participant presses a keystroke in response to a single stimulus and (2) a four-choice reaction time task where the participant presses a key corresponding to the correct response^38^. In this test, a participant is presented with multiple stimuli and asked to respond to one or more of them in a specific manner. The primary outcome of interest is the time taken between the presentation of the stimulus and the participant’s response, known as reaction time. Participants completed three trials of each test during each data collection time point.

### Gaming performance

We accessed game performance metrics through a publicly accessible website, *Tracker Network*, which is a third-party service that provides Valorant match statistics^39^. Participants create an account to join and allow the website to collect their match data. All participants were confirmed to have an account for this study. Matches played on Competitive and Swiftplay modes during the study duration were logged for each participant. Our analysis used kill/death ratio, headshot percentage, average damage per round, and average combat score as metrics for gaming performance.

### Statistical analysis

A priori power analysis was conducted using change in reaction time as the primary outcome variable. Our analysis was based on a prior study demonstrating improvements in reaction time after an intervention in esports players, with a mean difference of 0.01 s and a standard deviation of 0.01 seconds^40^. The alpha level was set at 0.05, with a desired power of 80%. With these parameters, the power analysis indicated that a sample size of 16 participants (each serving as their own control during the baseline period) was required. We repeated the power analysis using another primary outcome of hit/click accuracy, which was also studied in the above research by Sainz et al., and this yielded an identical sample size of 16 participants.

We performed normality testing of the sleep duration data using the Shapiro-Wilk test. Due to the non-normal distribution of our data and the repeated measurements for the same participants in both the pre- and post-intervention phases, we then used the Wilcoxon Signed rank test, a non-parametric test, to assess differences in sleep duration between both phases.

We created linear mixed-effects models to measure the differences in neurocognitive and gaming performance. Linear mixed-effects model analysis considers the repeated measures for each participant by including random intercepts for each subject, which allows the model to handle the within-subject correlation and variability across different time points. For our neurocognitive performance analysis, time point was treated as a fixed effect, while participants were included as a random effect to account for individual differences. The model can be expressed as Time_*ij*_ = *β*_0_ + *β*_1_⋅Timepoint_*ij*_ + *u*_*j*_ + $\epsilon_{ij}$ where Time_*ij*_ is the observed reaction time for individual *j* at time point *i*, *β*_0_ is the average reaction time at the baseline (when Timepoint = 0), *β*_1_ is the average change in reaction time at each subsequent time point, *u*_*j*_ is the random effect for each participant, and $\epsilon_{ij}$ is the residual error. Statistically significant results were then further analyzed using pairwise comparisons with linear mixed-effects model analysis to identify which pairs of time points showed significance. Statistical analyses and modeling of gaming parameters, including kill/death ratio, headshot percentage, average damage per round, and average combat score, were done similarly.

To evaluate HRV, we normalized each participant’s data on a common timeline from day 0 to 28 in both the pre-intervention and post-intervention periods. One of the participants did not have data recorded during the intervention phase; thus, 18 participants were included in the analysis. Data was then analyzed similarly above using a linear mixed-effects model to describe the effects of sleep intervention on HRV. The model was fitted to assess the impact of treatment condition, day, and sleep duration on HRV. Participants were included as a random effect. The model can be written as HRV*ij* = *β*_0_ + *β*_1_⋅Day_*ij*_ + *β*_2_⋅Group_*ij*_ + *β*_3_Sleep_*ij*_ + *u*_j_ + $\epsilon_{ij}$ where HRV*ij* is the observed HRV for individual *j* at timepoint *i*, *β*_0_ is the average HRV at baseline, *β*_1_ is the effect of day, *β*_2_ is the effect of treatment condition, *β*_3_ is the effect of sleep, *u*_j_ is the random effect for each participant, and $\epsilon_{ij}$ is the residual error.

Pearson correlation coefficients were calculated to evaluate the linear relationships between continuous variables. In our study, we compared the relationship between each neurocognitive test and Oura Ring average non-wear time. Correlation values range from − 1 to + 1 where values near ± 1 indicate strong linear relationships while values closer to 0 suggest weak or no linear association.

Statistical analyses were performed using Python (Version 3.12; Wilmington, Delaware, United States). The alpha level was set at 0.05. Results were considered statistically significant for *p* value < 0.05.

## Results

### Participant characteristics

A total of 20 individuals were eligible for participation (Table 1). One participant dropped out before the start of the study and was not included in the statistical analysis. The study sample consisted mostly of male undergraduate students with a mean age of 20.0 years (standard deviation (SD) = 1.6). All participants were high-performing athletes with Valorant game rankings at Diamond or above.

**Table 1:** Summary of participant demographics and gaming information. Measures are reported as percentages unless otherwise stated.

Category	Subcategory	Participant count (*n* = 19)	Proportion (%)
Sex	Male	16	84.2
	Female	3	15.8
Age	Mean (years) ± SD		20.0 ± 1.6
Education level	College student	18	94.7
	College graduate	1	5.3
Valorant rank	Diamond	4	21.1
	Ascendant	4	21.1
	Immortal	6	31.6
	Radiant	5	26.3
Hours of Valorant played per week	Mean (hours) ± SD		19.5 ± 13.5

### Sleep counseling on neurocognitive and gaming performance

**Fig. 1:**
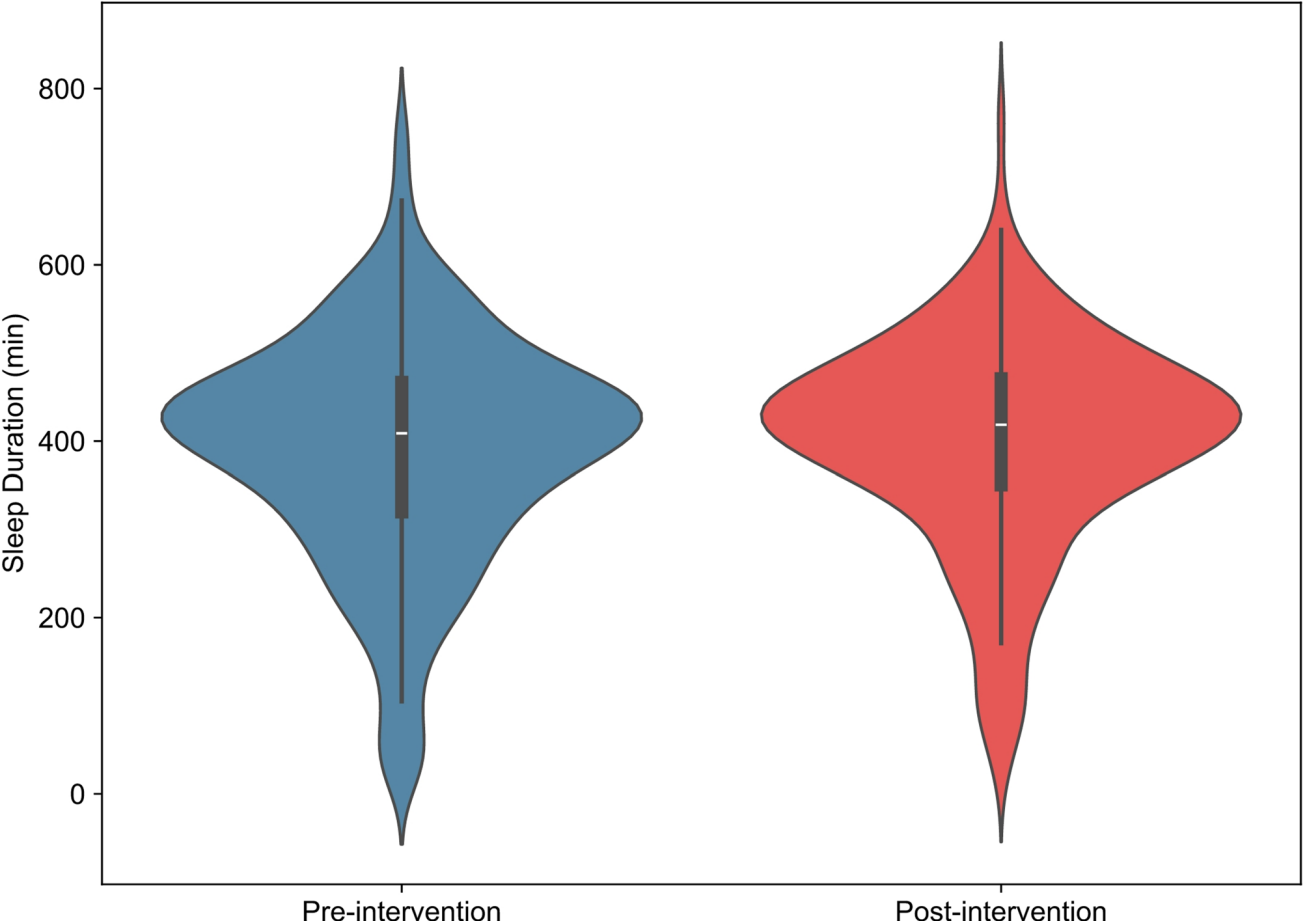
Representation of the average sleep duration (minutes) for all participants before and after sleep intervention.

We then evaluated if participants could significantly improve their sleep after the study’s midpoint (T_1_). We used daily sleep data collected from the Oura Ring for our analysis. Data was found to have a non-normal distribution. The pre-intervention sleep duration was 408.75 ± 6.17 min (median ± standard error) while post-intervention sleep duration was 418.50 ± 5.95 min (*p* = 0.265, Fig. 1). Overall, 61.1% of participants demonstrated an increase in sleep duration following the intervention.

Our study assessed neurocognitive performance using the PVT, SRT, and CRT at three different time points. For the PVT, the average reaction times decreased with each subsequent time point and were found to be statistically significant (*p* = 0.002). The SRT was not statistically significant (*p* = 0.313) and thus does not vary as a function of time. For the CRT, the estimated average test score at T_0_ was decreased with each time point. Similarly to the PVT performance, we observed statistically significant differences in the CRT between the time points (*p* = 0.001). We then performed pairwise comparisons to distinguish which specific periods showed significance based on the previously identified significant tests. For the PVT, there was a significant difference between T_0_ – T_2_ (*p* < 0.001) as well as T_0_ – T_1_ (*p* = 0.047) but not for T_1_ – T_2_ (*p* = 0.316). The significant difference between T_0_ – T_1_ suggests that the improvement is not attributable to any effects of sleep intervention and may be related to practice effects. For the CRT, there was a statistically significant difference between T_0_ – T_2_ (*p* < 0.001) but not between T_0_ – T_1_ (*p* = 0.052) or T_1_ – T_2_ (*p* = 0.127). A summary of the *p* values and pairwise comparisons are shown in Table 2.

**Table 2:** Linear mixed-effects model results for psychomotor vigilance test and simple and choice reaction time test throughout the study period.

	Mean difference between post-intervention vs. pre-intervention (ms)	95% confidence interval	*p* value	Pairwise comparison	Mean difference between post-intervention vs. pre-intervention (ms)	95% confidence interval	*p* value
Psychomotor Vigilance Test	−5.184	(−8.530, −1.839)	0.002^**^	T_0_ – T_1_	−7.281	(−14.466, −0.096)	0.047^*^
				T_0_ – T_2_	−10.368	(−16.060, −4.677	< 0.001^***^
				T_1_ – T_2_	−3.088	(−9.121, 2.945)	0.316
Simple Reaction Time Test	−2.526	(−7.435, 2.382)	0.313	T_0_ – T_1_	-	-	-
				T_0_ – T_2_	-	-	-
				T_1_ – T_2_	-	-	-
Choice Reaction Time Test	−7.947	(−12.634, −3.261)	0.001^**^	T_0_ – T_1_	−9.193	(−18.478, 0.092)	0.052
				T_0_ – T_2_	−15.895	(−25.251, −6.539)	< 0.001^***^
				T_1−_– T_2_	−6.702	(−15.320, 1.917)	0.127

^*^p < 0.05; ^**^p < 0.01; ^***^p < 0.001.

We also assessed individual gaming performance using in-game statistics such as kill/death ratio, headshot percentage, average damage per round, and average combat score at three different time points. The data were found to be non-normally distributed. Our findings showed no significant differences in individual gaming performance before and after sleep intervention (Table 3).

**Table 3:** Results of the linear mixed-effects models for each gaming performance metric.

	Mean difference between post-intervention vs. pre-intervention	95% confidence interval	*p* value
Kill/death ratio	−0.042	(−0.133, 0.049)	0.367
Headshot percentage	−0.914	(−1.871, 0.043)	0.061
Average damage per round	−0.410	(−5.450, 4.629)	0.873
Average combat score	−3.091	(−13.106, 6.923)	0.545

^*^p < 0.05; ^**^p < 0.01; ^***^p < 0.001.

### Effect of sleep counseling on heart rate variability

**Fig. 2:**
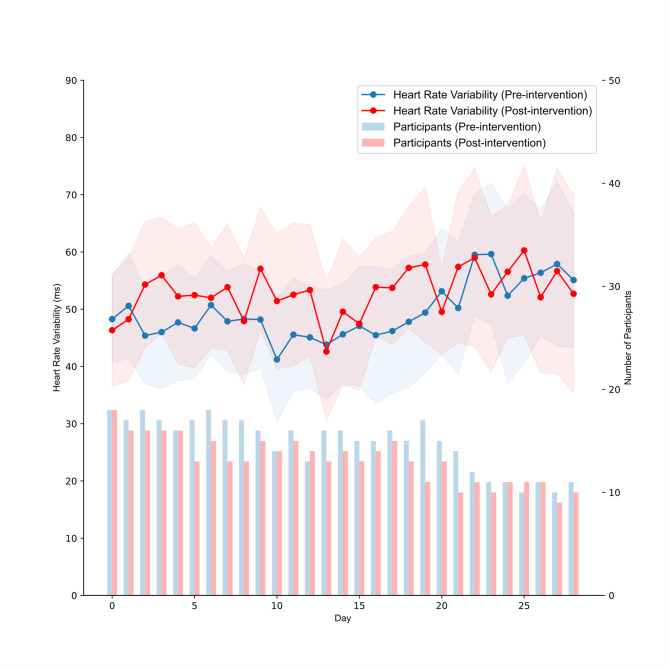
Aggregated heart rate variability over time for the pre-intervention and post-intervention phases, with a superimposed bar plot underneath showing the number of participants with data each day.

The Oura Ring calculates nighttime HRV using rMSSD, which has been shown to be a reliable indicator of autonomic nervous systemic activity. Each participant receives a daily average HRV value, which is a mean of all five-minute samples taken throughout the entire night. We examined the effect of sleep counseling on participant HRV and plotted changes over study period (Fig. 2). We found that HRV was significantly higher during the post-intervention phase than the pre-intervention phase (*p* < 0.05). To determine whether the impact of time and sleep was different between the pre-intervention and post-intervention periods, we next created a linear mixed-effects model that included interaction terms between the group and both time and sleep duration. This allowed us to evaluate whether the effect of time and sleep duration differed depending on the intervention type (Table 4). Both time and sleep duration positively affected HRV across the entire sample. However, their effects did not differ significantly between the pre-intervention and post-intervention phases. Thus, our final model excluded the interaction terms for simplicity.

95% confidence bands are shown for control and treatment data.

**Table 4:** Results of linear mixed-effects model for heart rate variability changes between pre-intervention and post-intervention phase.

Parameter	Mean difference between post-intervention vs. pre-intervention	95% confidence interval	*p* value
Group (intervention versus control)	2.849	(1.277, 4.421)	< 0.001^***^
Time (day)	0.123	(0.027, 0.219)	0.012^*^
Sleep duration (minutes)	0.015	0.009, 0.022)	< 0.001^***^

^*^p < 0.05; ^**^p < 0.01; ^***^p < 0.001.

### Wearable health device satisfaction

At each timepoint, participants were asked questions assessing their experience with the wearable health device. Responses were rated on a 5-point Likert scale, and the results are shown below (Table 5). Many participants rated overall quality highly, with 84.2% initially rating it as good or excellent; this increased to 94.7% over time. There were no very poor or poor responses. For battery life, 78.9% responded favorably, which increased to 84.9% at the study endpoint. Initially, no participants rated very poor or poor battery life, but this improved to 5.3% by the end of the study. The trend for ease of use of the device mobile app was the same as the trend for battery life. Regarding the comfortability of wearing the device, 84.2% of responses were good to excellent, which eventually increased to 89.4%. At the beginning of the study, 10.5% of participants rated device comfortability poorly, but this decreased to 5.4% by the study’s conclusion.

**Table 5:** Counts and proportions for survey questions regarding wearable device satisfaction throughout the study.

Time point	Question	Very poor	%	Poor	%	Average	%	Good	%	Excellent	%
T_0_	The overall quality of the device	0	0	0	0	3	15.8	7	36.8	9	47.4
	The battery life of the device	0	0	0	0	4	21.1	6	31.6	9	47.4
	The ease of use of the app	0	0	0	0	4	21.1	7	36.8	8	42.1
	The comfortability of wearing the device	0	0	2	10.5	1	5.3	10	52.6	6	31.6
T_1_	The overall quality of the device	0	0	0	0	1	5.3	11	57.9	7	36.8
	The battery life of the device	0	0	0	0	2	10.5	7	36.8	10	52.6
	The ease of use of the app	0	0	0	0	4	21.1	6	31.6	9	47.4
	The comfortability of wearing the device	0	0	1	5.3	3	15.8	7	36.8	8	42.1
T_2_	The overall quality of the device	0	0	0	0	1	5.3	10	52.6	8	42.1
	The battery life of the device	0	0	1	5.3	1	5.3	8	42.1	9	47.4
	The ease of use of the app	0	0	1	5.3	1	5.3	8	42.1	9	47.4
	The comfortability of wearing the device	0	0	1	5.3	1	5.3	10	52.6	7	36.8

### Wearable health device compliance

We observed that some participants had significant gaps in wearable data collection and explored the contribution of device non-compliance through post-hoc analysis. One of the many parameters the wearable health device can measure is device non-wear time. Data was normalized to each participant’s initial day of data collection, and average weekly non-wear time was calculated (Table 6). We found that the average non-wear time over the entire study for all participants was 297.7 min per week. When evaluating overall trends, we observed that 55.6% of participants had decreased non-wear time per day over the study duration. We then determined if those with lower non-wear time (i.e., compliant with the wearable device) had different outcomes than those with higher non-wear time. The Pearson correlation coefficients between average non-wear time and reaction times for the PVT, SRT, and CRT were 0.19, 0.16, and 0.24, respectively. These values indicate weak positive correlations and strength between non-wear time and neurocognitive test reaction times.

**Table 6:** Average weekly duration and standard deviation of non-wear time for all participants.

Week	Participant count	Average non-wear time (min)	Non-wear time standard deviation (min)
0	18	285.5	287.7
1	18	305.7	302.1
2	18	325.4	303.4
3	18	294.5	278.8
4	18	302.9	313.4
5	18	306.7	312.7
6	18	369.6	367.6
7	16	296.6	365.6
8	12	271.1	327.6
9	9	197.2	301.8
10	8	244.8	294.9
11	4	162.4	176.3

## Discussion

Our work investigates esports athletic performance, which has not been well studied previously. This gap in the literature requires further exploration as esports athletes are only able to infer strategies and decisions from research conducted in traditional sports disciplines. Prior studies suggest that sleep extension of approximately 45–120 min per night can significantly impact athletic performance^16,41,42^. In addition, studies on elite cricket athletes have shown that personalized sleep hygiene education can improve subjective sleep scores, sleep efficiency, sleep latency, and sleep onset variance^43^. This report presents our initial efforts to implement a sleep intervention and the challenges encountered.

### Sleep duration amongst esports athletes

In our analysis, esports athletes demonstrate no statistically significant increase in total sleep time compared to their baseline values. This suggests that the intervention was insufficient to overcome either ingrained behavioral patterns or external factors such as training or school schedules that limited opportunities for extended rest. It is also plausible that adherence to the sleep recommendations varied across participants or that the intervention was not reinforced strongly enough, minimizing any measurable effect on sleep duration. Initially, we hypothesized that both traditional sports and esports athletes experience similar sleep disturbances and would therefore benefit from the same strategies to improve rest. However, we should consider the possibility that the drivers of sleep impairment differ between these two groups. High physical training loads, travel-related jet lag, and pre-competition anxiety have all been linked to reduction in sleep quality and duration in traditional athletes^44^. In contrast, esports athletes face prolonged exposure to blue light from screens which disrupts alertness, melatonin secretion, and circadian timing that impact sleep quality (e.g., sleep latency) more than quantity^45,46^. Thus, sleep extension alone would be insufficient to correct the dysregulation seen in esports^47^; instead, targeted interventions (e.g., nutritional adjustments) aimed at improving sleep quality are warranted^48^.

### Neurocognitive and gaming performance outcomes

The PVT is widely used to assess sustained attention and alertness, particularly in sleep research. We anticipated no meaningful change in reaction times from the beginning to the midpoint of the study (i.e., baseline period, T_0_ – T_1_) and hypothesized that there would be a difference between the baseline and intervention period of the study. However, we observe a significant difference between the beginning and midpoint time points (T_0_ – T_1_) as well as the beginning and conclusion of the study (T_0_ – T_2_). There is no significant difference between midpoint and endpoint testing (T_1_ – T_2_). A possible explanation is that improvements were due to repeated exposure or familiarity with the task, otherwise known as practice effect, rather than actual changes in the underlying ability being measured^36,38^. This phenomenon is relevant in psychological and cognitive evaluations as individuals may perform better on subsequent administrations of the same or similar test simply because they have encountered the material before. Recognizing this effect is important when interpreting test outcomes.

We next assessed cognitive and motor function using the SRT, which primarily measures basic sensorimotor processing speed. We did not find significant differences in reaction time across study phases. Literature suggests that the SRT is less affected by external factors like fatigue and cognitive load^49^. As motor speed is relatively stable and less susceptible to variability compared to more complex cognitive tasks, our findings appear consistent with prior work. More challenging tasks are necessary to reveal sleep-related improvements in attention or decision-making. The SRT was complemented by the CRT, which assesses higher-level cognitive functions as it requires participants to perceive the stimulus and decide among multiple options before responding. We find significant differences in reaction time between the beginning and end of the study for the CRT (T_0_ – T_2_). Like the PVT, there is no significant difference between reaction times at the midpoint and the end of the study (T_1_ – T_2_). However, since the primary intervention did not lead to sleep extension, these changes cannot be interpreted as intervention effects.

Regarding gaming performance, our findings indicate no measurable difference before and after sleep counseling for our included metrics. Gaming performance is a complex combination of individual motor and cognitive skills as well as broader decision-making that can be affected by various external and internal factors^50^. The gaming parameters in our analysis could be influenced by unaccounted variables such as strategy, teamwork, map knowledge, and overall game flow, which may take some time to be affected by sleep improvement. These factors add additional complexity that may obscure the direct effect of sleep quality improvements. Future studies will be necessary to explore other contributing elements to provide a more comprehensive view of how sleep influences esports performance.

### Changes in heart rate variability over time

HRV measures the variation in time between consecutive heartbeats. It typically indicates autonomic nervous system function and reflects the heart’s ability to respond to physiological and environmental stimuli^51^. A high HRV suggests better physiologic adaptation to stress, whereas a low HRV indicates poor stress response. We find that day and sleep overall have positive associations with HRV, yet these effects remain similar across pre- and post-intervention periods, suggesting that the post-intervention phase does not confer a distinct advantage. Similar to our findings above, these changes should be interpreted with caution since we did not demonstrate sleep extension. More controlled work is needed to determine the usefulness of HRV for assessing adaptation and stress in esports athletes.

### Participant experience with a wearable health ring device

Although it remains the gold-standard sleep laboratory test, polysomnography requires an intensive setup with medical-grade equipment and can be both costly and time-consuming. Many professional sports teams have already incorporated wearable smart technology to assess athletes’ health and performance and monitor physiologic metrics, including sleep^52^. Our project was conducted on a small scale to identify the feasibility of using validated wearable technology as an alternative to polysomnography in a research setting. We included routine survey questions for participants to rate their overall experience with the PPG-based ring throughout the study. Participants report generally favorable satisfaction with this device, with most responses skewed towards higher ratings. Responses are largely consistent over time. Our findings align with prior research evaluating user satisfaction and experiences with various wearable devices^53^. Battery life has been shown as one of the most important barriers to consumer acceptance and usability, so this will be an important factor to consider when selecting an appropriate wearable device^54^. Notably, participants in our study rate battery life positively and consistently over time. While our study did not achieve the intended outcomes, it represents an important first step in applying wearable technology to sleep monitoring in esports athletes and establishes a practical protocol that can be scaled and refined in future investigations.

### Practical implications and future studies

Several key areas warrant further exploration to minimize bias and improve the reliability of results found in the study. We find statistically significant results in neurocognitive testing and HRV though the effect size was small. The numerical difference between pre- and post-intervention groups was small (often less than 10 ms); thus, the practical significance of such small changes remains uncertain. Without demonstrable sleep extension, the HRV differences cannot be causally linked to the study intervention. To our knowledge, there is no prior research assessing the performance relevance of sub-10ms improvements in the context of esports competition. Including subjective measures such as self-reported sleep quality (e.g., sleep diaries or Pittsburgh Sleep Quality Index questionnaire) in future research would provide better information on any discrepancies between how individuals perceive their sleep and how polysomnography would quantify it. Assessing the impact of other variables on gaming performance, such as physical activity, nutrition, team communication, and mental health, could provide a more holistic perspective on esports performance. For instance, individual performance in team-based settings can be influenced by teammate composition and even opponent behavior^55^. Future research should consider controlling for these variables (e.g., using fixed teammate lineups or standardized match conditions) to more accurately isolate the effects of individual treatment. Overall, our study demonstrates a well-structured approach to outcome measurement and achieved consistently high user satisfaction with the device. We hope our initial use of wearables for sleep monitoring in esports athletes inform future research aimed at improving athlete training.

### Limitations

The wearable PPG-based ring is a promising tool for collecting health data over time. Still, we encountered several limitations that could affect the generalizability and validity of our findings. First, missing data posed a significant challenge, as our study relied on a remote monitoring device, which prevented us from accurately assessing compliance with the study protocols. Several participants did not consistently wear the rings, resulting in gaps in data collection. However, variation in device non-wear time did not substantially bias or drive neurocognitive reaction times due to weak correlations for all three tests (*r* < 0.25). In addition, our study took place in an uncontrolled environment making it challenging to isolate the precise effects of our intervention. Potential confounding variables, such as varying sleep schedules, diet, and stress levels, could affect performance and HRV findings. There were also in-game variables we could not control, including interactions with other teammates (e.g., team dynamics and communication), equipment and internet speed, and players testing new strategies or characters. These variables may have influenced the outcomes but were not systematically accounted for in our research design. Another limitation was the lack of a separate control group in our study although this was due to the limited overall number of available and interested subjects. Since a traditional control group was not feasible, each participant served as their own control to allow us to compare baseline measurements to sleep intervention measurements. Future studies would benefit from including a larger study population to allow for a control group. Lastly, we did not control for practice or training effects in our serial neurocognitive assessments, which may have led to artificial improvements in performance unrelated to true cognitive change. Neurocognitive testing such as the PVT has been shown to be sensitive to practice effects, which could significantly limit data interpretation^36,38^. Future research should incorporate practice trials or alternate test versions to minimize error as described in previous work^56,57^. Despite these limitations, our study offers valuable insights into the potential use of wearable technology in monitoring and enhancing esports athletes’ performance and well-being.

## Conclusions

In conclusion, our study provides preliminary evidence highlighting the challenges involved with implementing sleep intervention in elite esports athletes. While improved reaction times and HRV were observed following sleep counseling, these effects may reflect secondary benefits rather than direct sleep extension, limiting causal inference. Despite this, our work demonstrates the feasibility of using wearables to investigate sleep and performance in esports athletes. Future research should further investigate how sleep influences gaming performance, neurocognitive function, and stress regulation, with the goal of enhancing both competitive outcomes as well as long-term physical and mental health.

## Supplementary Information

Below is the link to the electronic supplementary material.

Supplementary Material 1

## Data Availability

The datasets used and/or analyzed during the current study are available from the corresponding author on reasonable request.

## Abbreviations

PPG: Photoplethysmography
HRV: Heart rate variability
rMSSD: Root mean square of the successive differences
PVT: Psychomotor Vigilance Test
SRT: Simple Reaction Time Test
CRT: Choice Reaction Time Test
SD: Standard deviation
