# Supplementary material for: Exploring associations between sleep duration and performance as well as heart rate variability in elite esports athletes
Source: Sci Rep. 2025 Sep 24;15:32729. doi: 10.1038/s41598-025-18228-y (PMC12460822; doi:10.1038/s41598-025-18228-y)
Supplement: Supplementary file 1 — Supplementary Material 1 [file 41598_2025_18228_MOESM1_ESM.pdf]

UCI Esports Research Study – Improving Your Sleep Hygiene

Sleep hygiene is a term used to describe good sleep habits. There are many studies suggesting that such strategies can provide long-term solutions to sleep difficulties. The following are a list of tips for promoting sleep hygiene and healthy sleep habits.

- **Aim to increase your "Total Sleep" (which you can track in the Oura App) by at least 1 hour each night** (compared to your average "Total Sleep" over the past 1-2 weeks)
- Sleep and wake up at a regular and consistent time to maintain stable sleep cycles. Try to maintain this schedule even on days off or on the weekends
- Use the **Oura app** to help you gain a better understanding of how much and how well you are sleeping. The app will provide you with many metrics on the Sleep tab and identify areas of improvement. Consider using features within the app such as the Bedtime Guidance tool and library with tutorials which has resources such as information on sleep hygiene, relaxation, and breathing exercises
- Playing white noise or using ear plugs may help decrease occasional loud noises at night
- Consider adjusting room temperature to a comfortable temperature (if possible) as excessively warm or cold rooms can disturb sleep
- Hunger can disturb sleep so a light snack at least an hour before bedtime may help with sleep
- Melatonin is a natural hormone produced by your brain that helps with sleep. It often comes in gummies or tablets which may help with sleep
- Some people find performing a ritual before sleep to be helpful such as relaxing stretching or breathing exercises before bed
- Regular exercise can help promote good sleep; use the **Oura app** to track your activity levels over the next few weeks.
- Consider blocking out extra light with eye mask or curtains
- Avoid screen time (e.g., phones, computers, TV) in the 30-60 minutes before bedtime
- Avoid consuming alcohol, nicotine, and/or caffeinated products right before bedtime
- Avoid excessive liquids right before bedtime
- Avoid spending too much time awake in bed or doing activities in bed such as eating, studying, or watching TV
- Avoid napping during the day to ensure that you are tired at bedtime

Overall, the aim is to train and condition your body's biological clock to get consistent sleep. All the above suggestions may not help you fall asleep immediately tonight. However, we hope that incorporating even a few of the tips can help you start falling sleep faster and getting more restful sleep which will benefit you in your studies and gaming performance. Feel free to use the **Oura App** as much as you can, to get a better understanding of your sleep, readiness, and activities levels.
